# Influence of Positioning on Plain Levobupivacaine Spinal Anesthesia in Cesarean Section

**DOI:** 10.1155/2010/212696

**Published:** 2010-05-24

**Authors:** Fabio Gori, Francesco Corradetti, Vittorio Cerotto, Vito Aldo Peduto

**Affiliations:** Section of Anesthesia and Intensive Care, University of Perugia, 06100 Perugia, Italy

## Abstract

*Background*. The behaviour of isobaric levobupivacaine in relation to gravity when used in obstetric spinal anesthesia is unclear. *Methods*. 46 women with ASA physical status 1 undergoing cesarean section were randomly allocated to 2 groups. Spinal anesthesia with 12.5 mg levobupivacaine was performed in the sitting position in all women. Those in the first group were placed in the supine position immediately after the injection, while those in the second group were asked to remain seated for 2 minutes before assuming the supine position. The sensory block level, the onset of sensory and motor blocks, the regression of the sensory block for 2 dermatomes of the sensory block, the first request for analgesics, and the regression of motor block were recorded. *Results*. No differences in onset times, sensory level, or Bromage score were observed between the two groups. The time of first analgesic request was earlier in the seated group (supine 131 ± 42 min, seated 106 ± 29 min, *P* = .02). *Conclusion*. Isobaric levobupivacaine in women at term produces a subarachnoid block the dermatomal level of which does not depend on gravitational forces.

## 1. Introduction

Spinal anesthesia for cesarean section (CS) has gained popularity over epidural techniques because of its easy placement and rapid onset [1]. However, careful prevention of potential complications must always be sought to maintain a high safety profile. In pregnant women, engorgement of epidural veins from aortocaval compression with displacement of cerebrospinal fluid (CSF) may contribute to unwanted cephalad extensions of the block [2–4]. Furthermore, CS is a relatively short duration procedure that is often followed by early mobilization of the patient, which increases the potential for late extension of the block [5, 6].

The highest dermatomal level of analgesia from isobaric local anesthetics should be independent of patient position or gravity, but studies with isobaric bupivacaine in obstetrics have not demonstrated this [7–9] perhaps because isobaric bupivacaine is not truly isobaric. 

Levobupivacaine has a low systemic toxicity and, in addition, the plain solution has been shown to be truly isobaric with respect to CSF of pregnant women [10, 11]. Its use in this setting may therefore offer special advantages because this property may translate to a more predictable spread. On the basis of this evidence, we conducted a study to assess the variability of block extension in relation to the gravitational forces induced by a change in the position of the patient immediately following the injection of the anesthetic into the subarachnoid space.

## 2. Methods

We studied 50 American Society of Anesthesiologists (ASA) physical status 1 women who required elective CS for a delivery of a singleton baby at term. Our institutional Ethics Committee approved this study and all patients gave their written informed consent to participate. 

Patients were randomized to one of the two groups: early supine position or late supine position by computer-generated randomization schedule. Patients in the early supine position group (supine group) were placed in the supine position immediately after the placement of the spinal injection, while patients randomized to the second group (seated group) remained in the sitting position for 2 minutes before assuming the supine position. Exclusion criteria were contraindications to spinal anesthesia (coagulopathy, neuromuscular disease, and known allergy to local anesthetics).

Forty minutes before the induction of spinal anaesthesia we started the intravenous (i.v.) infusion of 1000 mL of lactated Ringer solution to provide volume preload. In both groups, spinal anesthesia was performed by one anesthesiologist using the same technique with the patient in the sitting position, using a midline approach at L3-L4 and a 27G Whitacre needle, and injecting 12.5 mg of isobaric levobupivacaine 0.5% over 30 seconds. Solutions were at room temperature (23°C). A wedge was placed under the right hip of the women during the spinal anesthesia procedure. 

Patients were monitored with continuous ECG and pulse oximetry and intermittent oscillometric blood pressure. Oscillometric blood pressure was measured every two minutes before extraction of the baby and every 5 minutes thereafter. The anesthesia was considered successful if the sensory block reached a T6 level in the first 20 minutes after the injection: if it was not, the spinal anesthesia was converted to general anesthesia with sevoflurane and the patient was withdrawn from the study. 

The onset phase of the sensory and motor blocks was monitored by pinprick tests and modified Bromage scores (0 = no paralysis, 1 = unable to raise extended leg, able to bend knee, 2 = unable to bend knee, able to flex ankle, 3 = no movement). These were repeated every two minutes until the start of surgery. 

If hypotension was detected, that is, a systolic pressure less than 90 mmHg or a reduction of 25% from baseline value, boluses of 2.5 mg ephedrine I.V. were administered as necessary. If the patient experienced pain or discomfort during surgery, alfentanil 0.1 mg/kg was administered as an intravenous bolus.

In the postoperative phase, vital signs and recovery dynamics (in the same way as during the onset) were checked every 30 minutes until complete regression of motor and sensory block had been attained. At each time point a Visual Analog Scale (VAS) (0–10 scale) for pain was administered, and when the score was found to be  >4, pain was controlled with morphine 10 mg i.m. and ketorolac 30 mg i.v. Time of first analgesic administration, time to regression of sensation by 2 dermatomes, time to recovery of sensory function at the L1 and S1 levels, and time to regression of the motor block were recorded. 

In the first 48 hours postoperatively analgesia was continued with ketorolac 30 mg i.v. every 8 hours. Acetaminophen 1 g I.v. was added 12 hours after the first administration of analgesics and then continued every 12 hours. During this period the patients were regularly checked for shivering, nausea, vomiting, respiratory depression, lumbar pain, and headache. 

Forty-two patients were needed to be included in the analysis in order to detect a difference between the two groups of 2 dermatomes in the sensory level reached during anesthesia, with an *α* error of 0.05 and power of 0.8. Interval data were compared with Students *t*-tests and frequencies with Fisher's exact test. Results are expressed as mean ± standard deviation. A *P* value of <.05 was considered significant.

## 3. Results

Each group consisted of 23 patients and demographics and clinical characteristics were similar between the two groups (Table 1). Two patients in each group did not reach the desired block level with spinal analgesia and were dropped from the study. Onset and recovery times are given in Table 2. Mean sensory dermatome level at the start and at the end of surgery was T5 in both groups; standard deviations (SD) were 0.67 and 0.57 dermatomes at the start of surgery and 0.97 and 0.71 dermatomes at the end of surgery for the supine and seated groups, respectively.

The Bromage score was not significantly different between the two groups at all checked time points, was less than 3 in five patients (2 in the supine group and 3 in the seated group, *P* = .8) before surgery, and was 3 in all patients after surgery. 

Times for first analgesic request were 131 ± 42 minutes and 106 ± 29 minutes (*P* = .02) in the supine and seated groups, respectively. 

Mean ephedrine dose was 5.8 ± 3.8 mg in the supine group and 4 ± 3.6 mg in the seated group and the difference was not statistically significant (*P* = .11). The number of patients requiring ephedrine was 19 and 17 in the supine and seated groups, respectively. The difference was not statistically significant (*P* = .72). Six patients in the supine group and 8 in the seated group required alfentanil (*P* = .74). No patient had headache, backache, paresthesia, shivering, or vomiting within 48 hours after surgery.

## 4. Discussion

Only a few studies have investigated obstetric spinal anesthesia using plain levobupivacaine. Parpaglioni et al., using an up/down sequential allocation method, determined that the ED50 for a satisfactory obstetric subarachnoid block is 10.6 mg [12]. Gautier et al., using a lower dose, found that levobupivacaine provided unsatisfactory results in 20% of patients, compared with 3% plain bupivacaine [13]. Studies in other types of surgery have given conflicting results. Burke et al., in a sample of 20 patients scheduled for lower limb surgery, found that anesthetic spread was unpredictable and widely dispersed among subjects [14]. Lee et al. reported good results in urologic surgery when compared to bupivacaine [15] both with and without addition of fentanyl [16], while Glaser et al. found no difference with bupivacaine in hip surgery [17]. 

In our study, the success rate (defined as an upper sensory level of T6) was 92%, which can be regarded as satisfactory. 

The density of cerebrospinal fluid in pregnant women is lower than that of men and nonpregnant women, and, according to accurate measurements, amounts to 1.00030 ± 0.00004 g/mL [11, 18]. 

The upper limit for hypobaricity is usually considered as −3 SD below this value, for example, 0.00018 g/mL [19]. Plain levobupivacaine 0,5% at 37°C has a density of 1.00024 ± 0.00009 g/mL [10] and could be considered isobaric. Theoretically, this characteristic should manifest itself as indifference to gravitational forces, applied both immediately after the injection and later on.

Our results confirm this hypothesis, demonstrating that levels of sensory block are unaffected by the position assumed in the first minutes following the injection. We studied two-minute sitting after spinal block since the combined spinal epidural is faster and easier in the seated patient but we did not know whether it was also safe and spare of the risk of saddle blocks in the occurrence of technical difficulties requiring the prolonged seated position.

Additionally, no significant differences were observed in onset times, Bromage score, recovery dynamics, and use of ephedrine. 

There is still some common belief that plain local anesthetics give unpredictable blocks, sometimes high, and that hyperbaric solutions are more reliable [1, 20] as they tend to spread to the thoracic kyphosis at approximately T4 when the patient lays down, regardless of patient height, and this pooling may facilitate a “one-dose-fits-all” approach [20]. 

Several reports show that plain bupivacaine has a tendency to give unexpectedly high levels of blocks, often after a position change and even after a reasonable time frame has been given to allow for fixation [21–23]. It is reasoned that all “plain” anesthetic solutions are actually hypobaric and tend to spread cephalad, causing these late complications [11]. 

Levobupivacaine may prove different in this respect, particularly in obstetric anesthesia, since its specific gravity is very close to that of the CSF of pregnant women [18].

Our study was powered to detect a difference in two dermatomes level of peak sensory block as we considered this to be a clinically significant difference. This is in contrast to our previous experience with *plain *bupivacaine,and current evidence. Ekeløf et al., evaluated the spread of 13.5 mg of*plain? * bupivacaine in obstetric spinals. That study showed a much wider distribution of sensory levels compared to our findings [24]. Similar results were found by Burke et al. [14] using levobupivacaine in a mixed population. We are still observing this phenomenon in our practice after the end of the study and believe that this is indeed caused by the “stillness” of plain levobupivacaine in the CSF of pregnant women. 

Although hyperbaric local anesthetic solutions have a remarkable record of safety, their use is not totally without risk: high spinals have been described with hyperbaric bupivacaine [25]. Their successful use requires rapid movement of the patient from the lateral or sitting position to prevent unilateral or saddle blocks, and conversely extension or return of the block may develop after mobilization [26–29]. Extension of the sympathetic block by the same mechanism may play a role in sudden cardiac arrest after spinal anesthesia with hyperbaric solution [5, 30]. Again, the use of truly isobaric solutions may prove less sensitive to position issues. This is very useful in a short procedure such as cesarean section where the hyperbaric local anesthetic that has not fixed [31] could migrate after early mobilization and cause hypotension or bradycardia.

The time of first analgesic request was statistically significantly different between the two groups with a mean difference of 25 minutes (supine 131 ± 42 minutes, seated 106 ± 29 minutes, *P* = .02), in contrast to supplemental analgesia needs during surgery that showed no difference whatsoever. The time for regression to L1 also showed a trend toward shorter times in the seated group. This difference is not readily explained and is possibly fortuitous.

An alternative hypothesis may be that the seated group experienced a slightly less dense block in the thoracic segments because of the known higher position of the spinal cord in the sitting position during the first minutes of anesthetic action. 

In conclusion, we did not find any influence of gravity on the spread of levobupivacaine in women undergoing spinal anesthesia for cesarean section. Moreover, the block levels were distributed in a relatively narrow range and the success rate was high, resulting in an overall good experience and a predictable anesthesia. Levobupivacaine may prove an excellent alternative to produce subarachnoid block for cesarean section.

## Figures and Tables

**Table 1:** Characteristics of the two groups.

	Seated group	Supine group	
	Mean (SD)	Mean (SD)	*P* value
Weight (Kg)	62.3 (13.7)	62.5 (16.8)	.15
Height (cm)	163 (6)	161 (5)	.22
Age (years)	32.9 (4.5)	33.5 (3.5)	.63
Gestational age (weeks)	38.3 (1.4)	37.8 (1.8)	.76
Duration of surgery (min)	42.6 (11.0)	40.7 (11.5)	.62

**Table 2:** Onset and recovery times.

	Seated group	Supine group	
	Mean (SD)	Mean (SD)	*P* value
Onset of sensory block (min)	7.2 (1.6)	7.6 (1.5)	.44
Onset of motor block (min)	6.4 (1.7)	5.6 (1.8)	.11
Two dermatomes regression (min)	80 (28)	76 (30)	.60
Regression to L1 (min)	176 (37)	158 (31)	.09
Regression to S1 (min)	241 (50)	230 (33)	.35
Complete motor recovery (min)	150 (48)	159 (34)	.46
First analgesic request	106 (29)	131 (42)	.02
